# Human corneal epithelial cell and fibroblast migration and growth factor secretion after rose bengal photodynamic therapy (RB-PDT) and the effect of conditioned medium

**DOI:** 10.1371/journal.pone.0296022

**Published:** 2023-12-27

**Authors:** Ning Chai, Tanja Stachon, Tim Berger, Zhen Li, Berthold Seitz, Achim Langenbucher, Nóra Szentmáry

**Affiliations:** 1 Dr. Rolf M. Schwiete Center for Limbal Stem Cell and Aniridia Research, Saarland University, Homburg, Saar, Germany; 2 Department of Ophthalmology, Saarland University Medical Center, Homburg, Saar, Germany; 3 Experimental Ophthalmology, Saarland University, Homburg, Saar, Germany; 4 Department of Ophthalmology, Semmelweis University, Budapest, Hungary; Massachusetts General Hospital, UNITED STATES

## Abstract

**Purpose:**

To investigate human corneal epithelial cell and fibroblast migration and growth factor secretion after rose bengal photodynamic therapy (RB-PDT) and the effect of conditioned medium (CM).

**Methods:**

A human corneal epithelial cell line (HCE-T), human corneal fibroblasts (HCF) and keratoconus fibroblasts (KC-HCF) have been used. Twenty-four hours after RB-PDT (0.001% RB concentration, 565 nm wavelength illumination, 0.17 J/cm^2^ fluence) cell migration rate using scratch assay and growth factor concentrations in the cell culture supernatant using ELISA have been determined. In addition, the effect of CM has been observed.

**Results:**

RB-PDT significantly reduced migration rate in all cell types, compared to controls (p≤0.02). Migration rate of HCE-T cultures without RB-PDT (untreated) was significantly higher using HCF CM after RB-PDT, than using HCF CM without RB-PDT (p<0.01). Similarly, untreated HCF displayed a significantly increased migration rate with HCE-T CM after RB-PDT, compared to HCE-T CM without treatment (p<0.01). Furthermore, illumination alone and RB-PDT significantly decreased keratinocyte growth factor (KGF) concentration in HCF and KC-HCF supernatant, and RB-PDT significantly decreased soluble N-Cadherin (SN-Cad) concentration in HCF supernatant, compared to controls (p<0.01 for all). In HCE-T CM, RB-PDT increased hepatocyte growth factor (HGF) and basic fibroblast growth factor (FGFb) concentration (p≤0.02), while decreasing transforming growth factor β (TGF-β) concentration (p<0.01). FGFb concentration increased (p<0.0001) and TGF-β concentration decreased (p<0.0001) in HCF CM, by RB-PDT. Epidermal growth factor (EGF), HGF, and TGF-β concentration decreased (p≤0.03) and FGFb concentration increased (p<0.01) in KC-HCF CM, using RB-PDT.

**Conclusions:**

HCE-T, HCF and KC-HCF migration rate is reduced 24 hours after RB-PDT. In contrast, HCE-T migration is enhanced using HCF CM after RB-PDT, and HCF migration rate is increased through HCE-T CM following RB-PDT. Modulation of EGF, KGF, HGF, FGFb, TGF-β and N-Cadherin secretion through RB-PDT may play an important role in corneal wound healing.

## 1. Introduction

Photodynamic therapy (PDT) became a potential treatment alternative in different types of diseases, in the last decades [1–3]. During PDT, different photosensitizers, such as riboflavin, Chlorin e6, rose bengal (RB) or 5-aminolevulinic acid and light of an appropriate wavelength are used [4–7].

Due to its superficial location and transparency, the corneal tissue may also undergo PDT. Crosslinking (CXL), as riboflavin-UVA-PDT was first introduced by Spoerl et al in 1998 [1]. CXL induces cross-links between the collagen fibers of the corneal tissue increasing corneal stiffness and stopping progression of keratoconus [8]. In addition, CXL also has promising applications in treatment of corneal melting, infectious keratitis, corneal ulcer and bullous keratopathy [9–13].

Rose bengal photodynamic therapy (RB-PDT), using green light (maximal absorption wavelength: 550–560 nm) may also be a promising treatment method in several ophthalmic pathologies [14, 15]. RB-PDT seemed to be an effective corneal stiffening procedure using animal models [16, 17]. Furthermore, using RB-PDT, some patients with infectious keratitis successfully healed [8, 15].

Although RB-PDT has already been used in patients with corneal pathologies, the effect of RB-PDT on corneal epithelial cells and fibroblasts has not been fully understood, yet. In the current study, our aim was to investigate human corneal epithelial cell and human corneal fibroblast migration and their growth factor secretion after RB-PDT, and the epithelial-stromal interaction through detecting the effects of conditioned medium (CM).

## 2. Materials and methods

This study was approved by the Ethics Committee of Saarland/Germany (Nr. 217/18). All human tissues were handled according to the Declaration of Helsinki principles.

### 2.1 Experimental reagents

**Dulbecco’s modified Eagle’s medium (DMEM/F12)** (Thermo Fisher Scientific, Waltham, MA, USA); 5% fetal calf serum **(FCS)** (Thermo Fisher Scientific, Waltham, MA, USA); 1% penicillin-streptomycin **(P/S)** (Sigma-Aldrich, St. Louis, USA); **Collagenase A** (Hoffmann-La Roche, Basel, Switzerland); **Trypsin EDTA** (Sigma-Aldrich, St. Louis, USA); **Epidermal Growth Factor** (EGF, Gibco, Paisley, UK); **Glutamine** (Sigma Aldrich, Germany); 1% **insulin, transferrin, and selenium (ITS)**; **PBS** (Merck, Sigma-Aldrich, Taufkirchen, Germany); **Rose Bengal B** (C.I. 45440, Carl Roth, Karlsruhe, Germany); **RIPA buffer** (Thermo Fisher Scientific, Waltham, MA, USA).

### 2.2 Human corneal epithelial cell line (HCE-T)

The used human corneal epithelial cell line (HCE-T) was provided by the RIKEN cell bank (RCB 2280, Ibaraki, Japan). HCE-T cells were cultured in DMEM/F12 medium, supplemented by 5% FCS, 1% P/S, 10 ng/ml EGF and 1% ITS (“*HCE-T medium”*), using T75 flasks. After reaching 80% of confluence, HCE-T cells were detached using trypsin EDTA and were seeded into 6-well plates for the subsequent experiments.

### 2.3 Human corneal fibroblast (HCF) and human keratoconus corneal fibroblast (KC-HCF) isolation

Five healthy human corneoscleral buttons were provided by the Klaus Faber Center for Corneal Diseases, including Lions Eye Cornea Bank Saar-Lor-Lux, Trier/Westpfalz, in Homburg. These tissues didn’t meet the requirements for corneal transplantation, due to low endothelial cell density (<1800 cell/cm^2^). Five human keratoconus corneal samples were obtained from elective penetrating keratoplasties (all patients signed an informed consent, before surgery). The donor tissues / keratoconus samples were first rinsed by PBS, then were cut into small tissue pieces through a surgical scalpel (tissue diameter about 5 mm). Then, the small tissue pieces were put into a solution containing 1.0 mg/ml collagenase A, supplemented by DMEM/F12, 5% FCS, and 1% P/S. After incubation at 37°C overnight, centrifugation has been performed at 1500 rpm for 5 minutes, and the supernatant was discarded. Thereafter, the cell sediment was resuspended in 1 ml DMEM/F12, supplemented by 5% FCS and 1% P/S (“*basic medium*” in the following text) and was seeded into a T75 flask, containing 13 ml basic medium. The cell containing flasks were subsequently stored at 37°C, using 5% CO_2_, and 95% relative humidity in the incubator. The medium was changed every 4 days. As soon as HCF and KC-HCF reached 80% confluence, these were harvested by trypsin EDTA and were passaged to 8 new T75 flasks. After reaching 80% confluence, HCF and KC-HCF were detached using trypsin EDTA and were seeded into 6-well plates for the subsequent experiments.

### 2.4 RB-PDT

The RB stock powder was stored in a dark environment until use. As the first step, the 0.001% (m/v) RB solution was prepared by dissolving the stock powder in *HCE-T* or *basic medium*. After sterilization by a 0.2 μm sterilizing filter, the RB solution was stored at 4°C in darkness, for up to 1 month.

The illumination box was built and calibrated at the Department of Experimental Ophthalmology, Saarland University, Homburg/Saar, Germany. In the present study, besides using 0.001% RB concentration, 565 nm wavelength illumination has been used and illumination time was always 600 s. During the present measurement series, we used 0.17 J/cm^2^ fluence for all cell types. The used fluence values have been chosen based on our previous measurement series, which analyzed cell viability after RB-PDT *in vitro* [3]. The actually used fluence values were the lowest values, which already slightly affected cell viability.

When HCE-T, HCF or KC-HCF reached 80% confluence in the 6-well plates, treatment has been performed, using the following groups:

1. The cells in the control group (Ctrl) have been gently rinsed three times with 3 ml PBS, without the use of RB or illumination. Then, 2 ml DMEM/F12 supplemented by 1% ITS and 1% P/S has been added to each well.
2. The cells in the “RB only” group have been allowed to absorb the 0.001% RB solution for 30 minutes at 37°C without further light illumination, which has been followed by gentle rinsing three times with 3 ml PBS. Then, 2 ml DMEM/F12 supplemented by 1% ITS and 1% P/S has been added to each well.
3. The cells in the “illumination only” group have been rinsed two times with 3 ml PBS, then 3 ml PBS has been added to each well and the cells have been illuminated by green light using 0.17 J/cm^2^ fluence, without adding RB. Thereafter, PBS was replaced by 2 ml DMEM/F12 supplemented by 1% ITS and 1% P/S.
4. The cells in the RB-PDT group have been allowed to absorb the 0.001% RB solution for 30 minutes at 37°C, which has been followed by rinsing with 3 ml PBS twice. Thereafter, 3 ml PBS has been added to each well and the cells have been subsequently illuminated using 0.17 J/cm^2^ fluence. Then, PBS was replaced by 2 ml DMEM/F12 supplemented by 1% ITS and 1% P/S.

### 2.5 HCE-T, HCF and KC-HCF migration rate after treatment

As the first step, three parallel reference lines have been drawn at the bottom of the wells of the 6-well plates with 5 mm distance. Then, HCE-T, HCF or KC-HCF have been seeded, using 15000 cells/cm^2^ and adding 3 ml HCE-T medium or basic medium. Reaching 80% confluence, one scratch has been generated in each well, perpendicular to the drawn bottom reference lines, using 100 μl pipette tips (Eppendorf AG, Hamburg, Germany). Then, treatment has been performed as described above (groups (1) to (4)).

Phase-contrast images have been taken directly after treatment (0 hour) and 24 hours afterwards. For each well, 4 different scratch areas have been photographically documented, along the previously drawn reference lines (**S1 Fig**), for subsequent analysis.

Images of 3 independent scratch assay experiments using HCE-T cell line and images of 5independent scratch assay experiments using HCF (from 5 different donors) and KC-HCF (from 5 different keratoconus corneas) have been collected.

Phase contrast images have been analyzed using ImageJ (https://imagej.nih.gov/ij/). The Migration Rate (MR) has been calculated as follows:

MR = [Wound area (0 h)–Wound area (24 h)] / Wound area (0 h).

**Fig 1:**
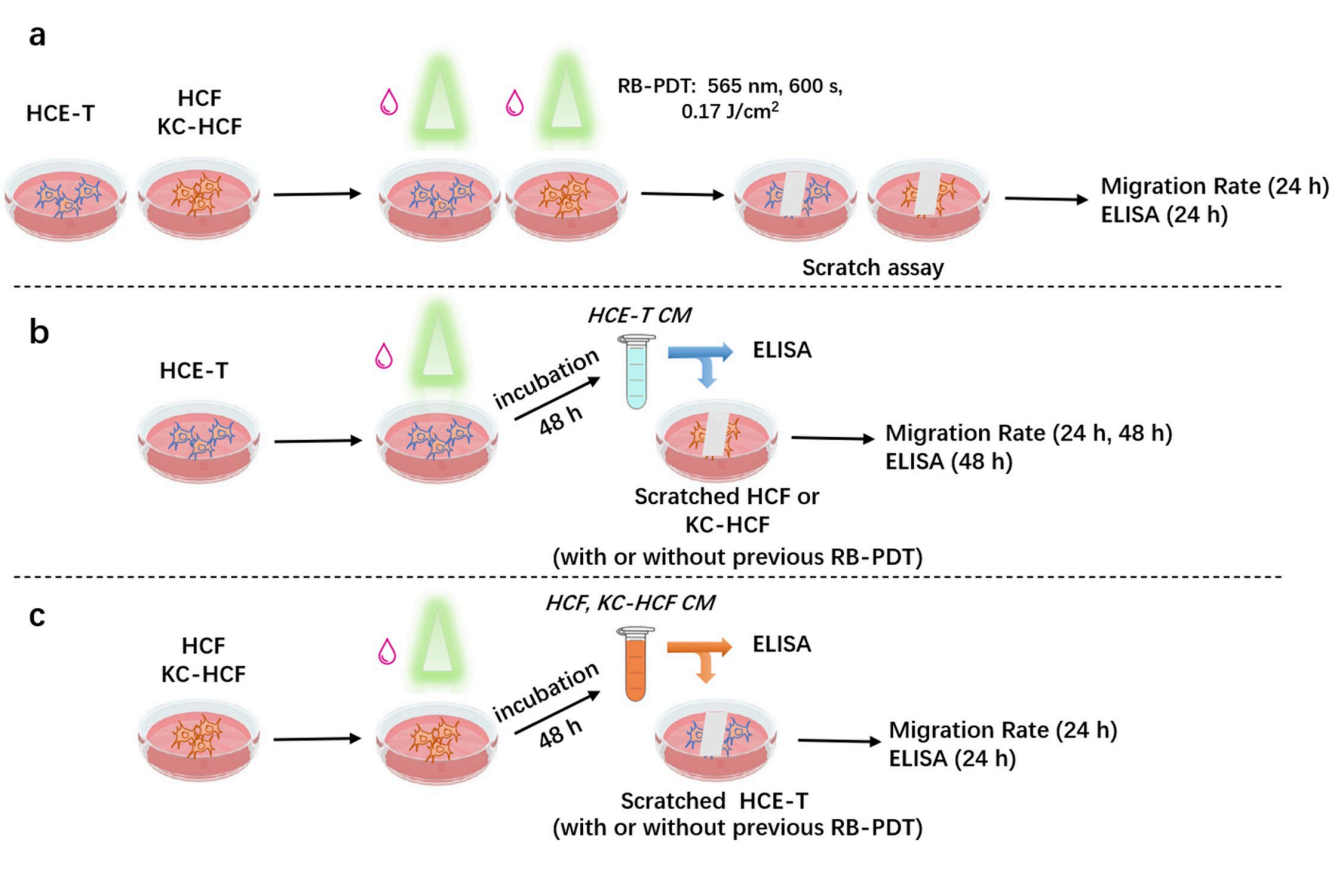
Schematic illustration of the experiments. (a) Human corneal epithelial cells (HCE-T), human corneal fibroblasts (HCF) and human keratoconus fibroblasts (KC-HCF) underwent rose bengal photodynamic therapy (RB-PDT; 0.001% RB concentration, 565 nm wavelength illumination, 17 J/cm^2^ fluence), which was followed by scratch assay (migration rate measurement). Thereafter, the supernatant has been collected for the Enzyme-Linked Immunosorbent Assay (ELISA). (b) HCE-T underwent RB-PDT to generate HCE-T conditioned medium (CM). This CM has been added to scratched HCF and KC-HCF cultures to observe its effect on migration rate. The CM has also been collected for ELISA. (c) HCF and KC-HCF underwent RB-PDT to generate HCF or KC-HCF CM. This CM has been added to scratched HCE-T cultures to observe its effect on migration rate. The CM has also been collected for ELISA.

Schematic description of our experiments is shown at **Fig 1**.

### 2.6 HCE-T migration rate using HCF or KC-HCF conditioned medium

In order to observe epithelial cell (HCE-T) and stromal cell (HCF or KC-HCF) interactions after treatment, conditioned medium (CM) has been used. For these experiments, only in the “RB-PDT” chapter described “Ctrl” (1) and “RB-PDT” (4) groups have been included.

In brief, HCF and KC-HCF underwent treatment, as described above (only “Ctrl” (1) and “RB-PDT” (4) groups). As the last step of the treatment, 2 ml DMEM/F12 supplemented by 1% ITS and 1% P/S has been added to each well. After *48 hours* at 37°C, the HCF or KC-HCF supernatant has been collected from each well. Cell remnants have been removed from the supernatant using a centrifugation step (3000 rpm for 4 minutes) to get the subsequently used HCF or KC-HCF conditioned medium (HCF CM or KC-HCF CM).

HCE-T scratches have been performed as described above and HCE-T underwent treatment (only “Ctrl” or “RB-PDT” groups). However, instead of adding 2 ml DMEM/F12 supplemented by 1% ITS and 1% P/S as the last step of treatment, HCE-T cultures have been supplemented by the above described 2 ml HCF or KC-HCF CM and were incubated for 24 hours at 37°C. Conditioned media have not been pooled, these have been used separately for each well.

Scratch assay images from each treatment group have been collected, as described above directly after treatment (0 hour) and after 24 hours.

### 2.7 HCF or KC-HCF migration rate using HCE-T conditioned medium

For these experiments, also only in the “RB-PDT” chapter described “Ctrl” (1) and “RB-PDT” (4) groups have been included.

In brief, HCE-T underwent treatment, as described above (only “Ctrl” (1) and “RB-PDT” (4) groups). As a last step of the treatment, 2 ml DMEM/F12 supplemented by 1% ITS and 1% P/S has been added to each well. After *48 hours* at 37°C, the HCE-T supernatant has been collected from each well. Cell remnants have been removed from the supernatant using a centrifugation step (3000 rpm for 4 minutes) to get the subsequently used HCE-T conditioned medium (HCE-T CM).

HCF and KC-HCF scratches have been performed as described above and HCF and KC-HCF underwent treatment (only “Ctrl” or “RB-PDT” groups). However, instead of adding 2 ml DMEM/F12 supplemented by 1% ITS and 1% P/S as the last step of RB-PDT, HCF and KC-HCF cultures have been supplemented by the above described 2 ml HCE-T CM and were incubated for up to 48 hours at 37°C. Conditioned media have not been pooled, these have been used separately from each well.

Scratch assay images from each treatment group have been collected, as described above directly after treatment (0 hour) and after 24 and 48 hours.

### 2.8 Enzyme linked immunosorbent assay (ELISA) of the cell culture supernatant

Epidermal growth factor (EGF), keratinocyte growth factor (KGF), hepatocyte growth factor (HGF), basic fibroblast growth factor (FGFb) and transforming growth factor β (TGF-β) concentrations in HCE-T, HCF and KC-HCF culture supernatant 24 hours after treatment and scratching have been measured using ELISA (Groups (1) to (4)). In addition, EGF, HGF and KGF concentration has been measured in 48-hours-conditined HCE-T CM, HCF and KC-HCF CM (without scratch) and in the supernatant of scratched HCE-T, HCF and KC-HCF cultures, 24 or 48 hours after addition of different CM.

To perform the ELISA measurements, DuoSet^®^ ELISA kits (Epidermal growth factor, EGF: DY251; Keratinocyte growth factor, KGF: DY251; Hepatocyte growth factor, HGF: DY294; Basic fibroblast growth factor, FGFb: DY233; Transforming growth factor β, TGF-β: DY240; E-Cadherin: DY648; N-Cadherin: DY1388-05) were purchased from R&D Systems (Minneapolis, USA). All measurements were performed in duplicate. The measurement was performed according to the manufacturer’s protocol. In brief, 100 μl capture antibody was added to 96-well plates, and these were incubated overnight at room temperature. Thereafter, 100 μl supernatant was added to each well for 2 h. This step was followed by incubation with the detection antibody for another 2 h. The optical density (OD) value of the wells was detected using a Tecan Infinite F50 Absorbance Microplate Reader (Tecan Group AG, Männedorf, Switzerland) in order to assess growth factor concentration quantitatively. The possessed value was divided by the total protein concentration of the well to obtain the concentration in pictogram (pg) per milligram protein (mg). This value was used for statistical analysis.

### 2.9 Statistical analysis

Data have been analyzed using Graphpad Prism version 9.2 (GraphPad Software, San Diego, CA). Data were expressed as mean ± standard error of the mean (SEM). In order to compare multiple groups, one-way ANOVA followed by Dunnett’s multiple comparison test was used and in order to compare two groups, the unpaired student’s t-test was used. P values below 0.05 were considered statistically significant.

## 3. Results

### 3.1 HCE-T, HCF and KC-HCF migration rate after treatment

**Fig 2:**
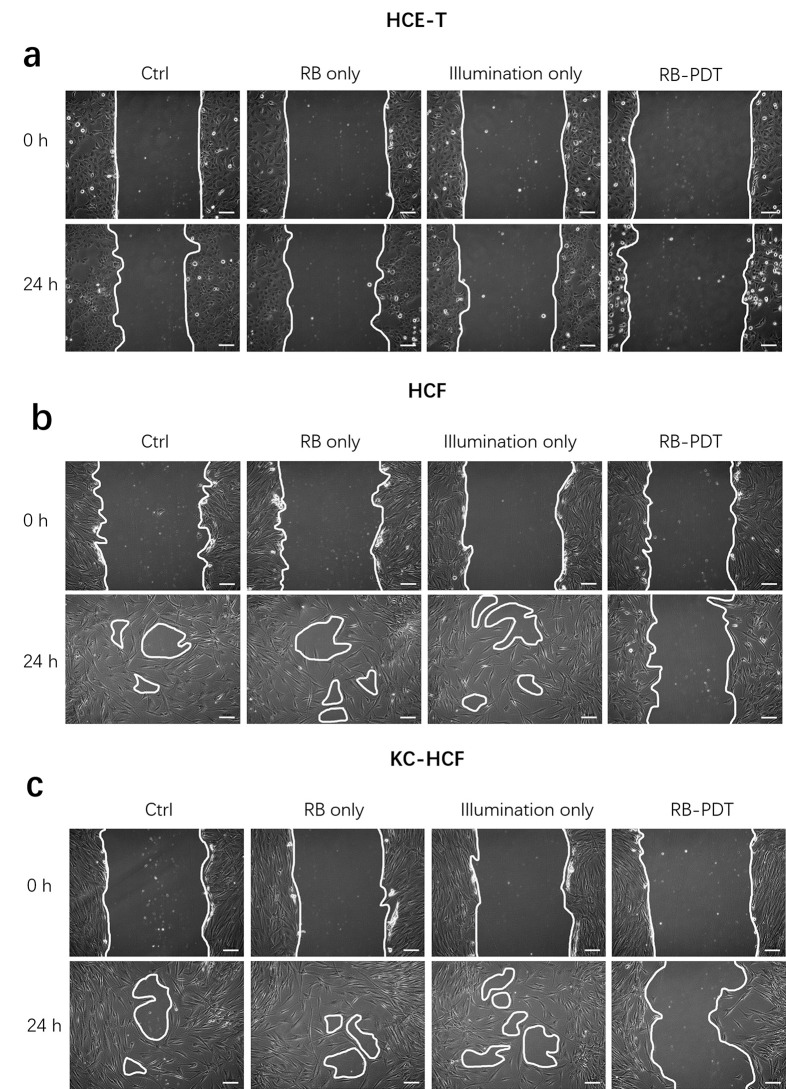
Representative phase contrast images of human cornea epithelial cell (HCE-T). (a), human corneal fibroblast (HCF) (b) and human keratoconus fibroblast (KC-HCF) (c) cultures directly after scratching (0 h) and 24 hours after treatment (Scale bar: 50 μm).

**Fig 3:**
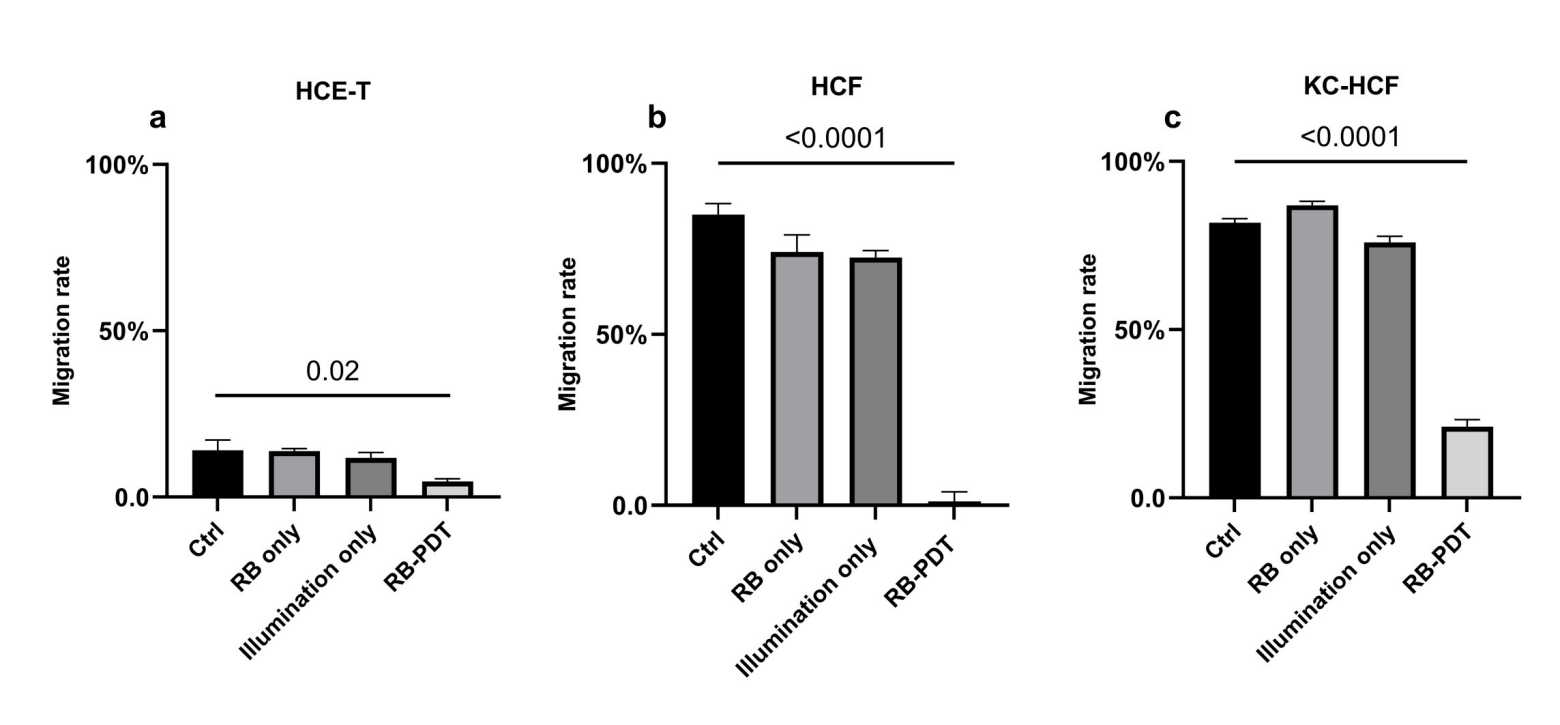
Migration rate in human corneal epithelial cell line (HCE-T) (n = 3), human corneal fibroblast (HCF) (n = 5) and human keratoconus fibroblast (KC-HCF) (n = 5) cell cultures, 24 hours after treatment and scratching. Data are expressed as mean ± SEM. 0.17 J/cm^2^ rose bengal photodynamic therapy (RB-PDT) significantly decreased migration rate in all cell types, compared to controls (Ctrl) (p≤0.02). The experiments using primary human corneal fibroblasts (HCF, KC-HCF) were repeated five times (from five different donors), while the experiments using a commercial corneal epithelial cell line (HCE-T) were repeated three times.

**Fig 2** displays phase contrast images of HCE-T, HCF and KC-HCF cultures, directly after scratching (0 h) and 24 hours after treatments. **Fig 3** shows migration rate in the same cultures, 24 hours after treatment. 0.17 J/cm^2^ RB-PDT significantly decreased migration rate in all cell types, compared to Ctrl (p≤0.02). Nevertheless, using RB only, or illumination only, migration rate did not change in any of the cell types.

### 3.2 Growth factor concentration in the cell culture supernatant, after treatment and scratching

**Fig 4:**
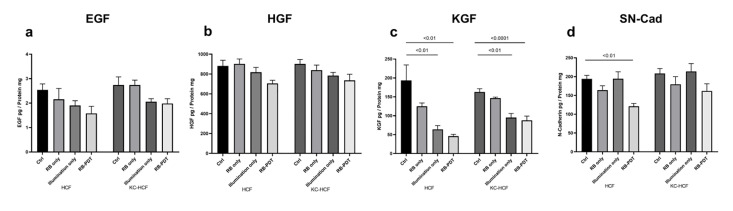
Epidermal growth factor (EGF), hepatocyte growth factor (HGF), keratinocyte growth factor (KGF), Soluble N-Cadherin (SN-Cad) concentration in human corneal fibroblast (HCF) and human keratoconus fibroblast (KC-HCF) cell culture supernatant 24 hours after treatment and scratching, as assessed by ELISA (n = 5). Data are expressed as mean ± SEM. 0.17 J/cm^2^ illumination alone and 0.17 J/cm^2^ rose bengal photodynamic therapy (RB-PDT) significantly decreased KGF concentration in both cell types, compared to controls (Ctrl) (p<0.01). In addition, 0.17 J/cm^2^ RB-PDT, significantly decreased SN-Cad concentration in HCF cultures, compared to controls (p<0.01).

Growth factor and soluble N-Cadherin (SN-Cad) concentrations in HCF and KC-HCF culture supernatant 24 hours after treatment and scratching are displayed at **Fig 4**. EGF, HGF and KGF concentration was not measurable in HCE-T cell culture supernatant 24 hours after treatment and scratching. FGFb and TGF-β concentrations were also not measurable in HCE-T, HCF and KC-HCF cell culture supernatant 24 hours after treatment and scratching. 0.17 J/cm^2^ illumination alone and 0.17 J/cm^2^ RB-PDT significantly decreased KGF concentration in HCF and KC-HCF cell culture supernatant, compared to controls (p<0.01) (**Fig 4C**). Nevertheless, EGF and HGF concentration, and KGF concentration in the RB only group did not differ significantly from other treatment groups 24 hours after treatment and scratching (**Fig 4A–4C**). Furthermore, 0.17 J/cm^2^ RB-PDT, significantly decreased SN-Cad concentration in HCF cultures, compared to controls (p<0.01).

### 3.3 HCE-T migration rate using HCF or KC-HCF conditioned medium

**Fig 5:**
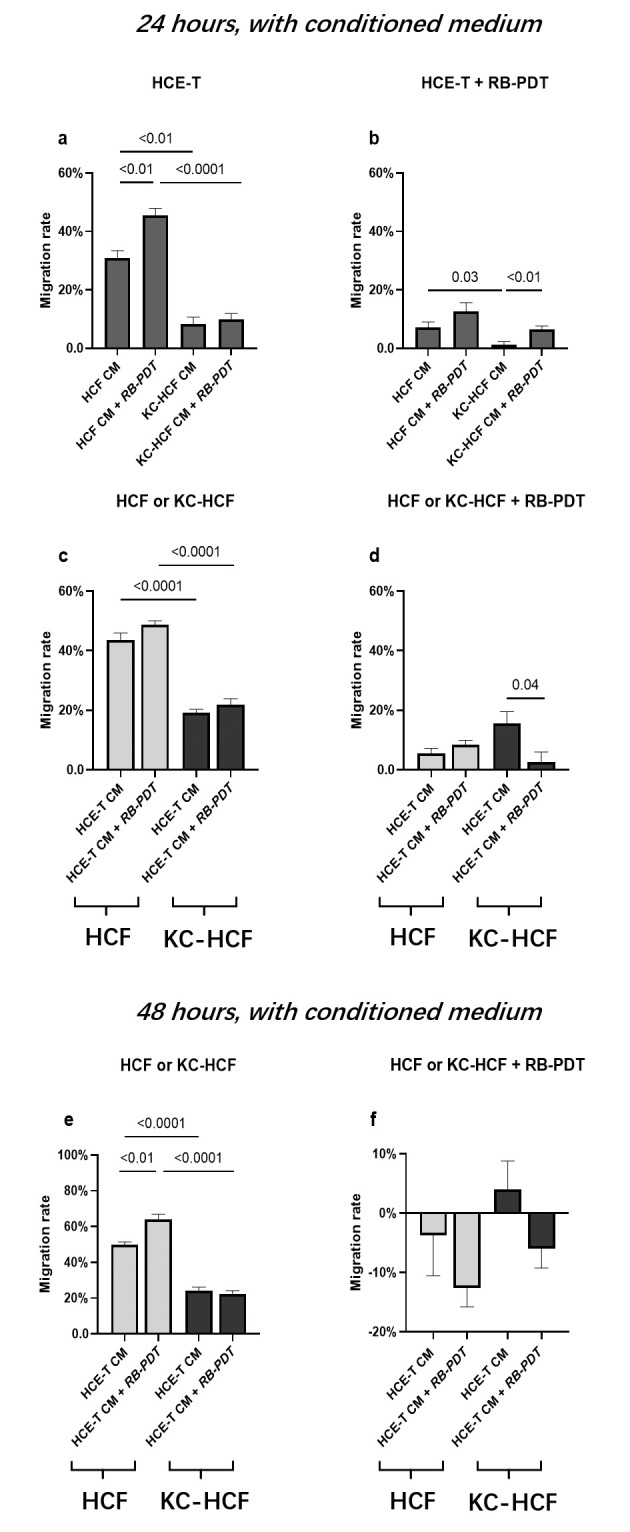
Migration rate in untreated and treated by rose-bengal photodynamic therapy (RB-PDT) human corneal epithelial cell (HCE-T) (n = 3), human corneal fibroblast (HCF) (n = 5) and human keratoconus fibroblast (KC-HCF) (n = 5) cell cultures after 24 and 48 hours, using conditioned medium (CM). Migration rate of untreated HCE-T cultures was significantly higher using HCF-CM after RB-PDT, than using HCF-CM without RB-PDT (p<0.01, 5a). Migration rate of untreated HCF was significantly higher using HCE-T CM after RB-PDT, than using HCE-T CM without RB-PDT (p<0.01, 5e). 48 hours after the use of CM, migration rate of RB-PDT treated HCF cultures using HCE-T CM with or without RB-PDT, and in KC-HCF cultures, using HCE-T CM after RB-PDT was in a negative range (although the wound area did not increase significantly compared to baseline) (5f). Data are expressed as mean ± SEM. HCE-T CM: 48 hours conditioned HCE-T cell culture supernatant without or with previous rose bengal photodynamic therapy (RB-PDT), without scratch. HCF CM/KC-HCF CM: 48 hours conditioned HCF/KC-HCF cell culture supernatant without or with previous rose bengal photodynamic therapy (RB-PDT), without scratch.

HCE-T migration rate after 24 hours, using HCF or KC-HCF CM is displayed at **Fig 5A and 5B**.

Migration rate of untreated HCE-T cultures was significantly higher using RB-PDT treated HCF CM, than using HCF CM without RB-PDT (p<0.01) (**Fig 5A**. Migration rate of untreated HCE-T cultures was also significantly higher using HCF CM, than with KC-HCF CM (p<0.01). In addition, migration rate of untreated HCE-T cultures was also significantly higher using HCF CM after RB-PDT, than with KC-HCF CM after PDT (p<0.0001) (**Fig 5A**).

Migration rate of RB-PDT treated HCE-T cultures was significantly higher using HCF CM, than using KC-HCF CM (p = 0.03) (**Fig 5B**). In addition, migration rate in RB-PDT treated HCE-T cultures was significantly higher using KC-HCF CM after RB-PDT, than using KC-HCF CM without RB-PDT (p<0.01) (**Fig 5B**).

### 3.4 HCF and KC-HCF migration rate using HCE-T conditioned medium

HCF and KC-HCF migration rate after 24 and 48 hours, using HCE-T CM is displayed at **Fig 5C–5F**.

Migration rate was significantly higher in HCF, than in KC-HCF after 24 hours, using HCE-T CM (p<0.0001). Migration rate was also significantly higher in HCF, than in KC-HCF after 24 hours, using RB-PDT treated HCE-T CM (p<0.0001) (**Fig 5C)**.

In RB-PDT treated KC-HCF cultures, migration rate after 24 hours was significantly higher using HCE-T CM without RB-PDT, than using HCE-T CM after PDT (p = 0.04) (**Fig 5D)**.

Migration rate was significantly higher in HCF, than in KC-HCF, after 48 hours, using HCE-T CM (p<0.0001). Migration rate was also significantly higher in HCF, than in KC-HCF after 48 hours, using RB-PDT treated HCE-T CM (p<0.0001). In addition, in HCF cultures, migration rate after 48 hours, was significantly higher using HCE-T CM with RB-PDT, than using HCE-T CM without PDT (p<0.01) (**Fig 5E)**.

### 3.5 Growth factor concentration in 48 hours conditioned medium, without scratching

**Fig 6:**
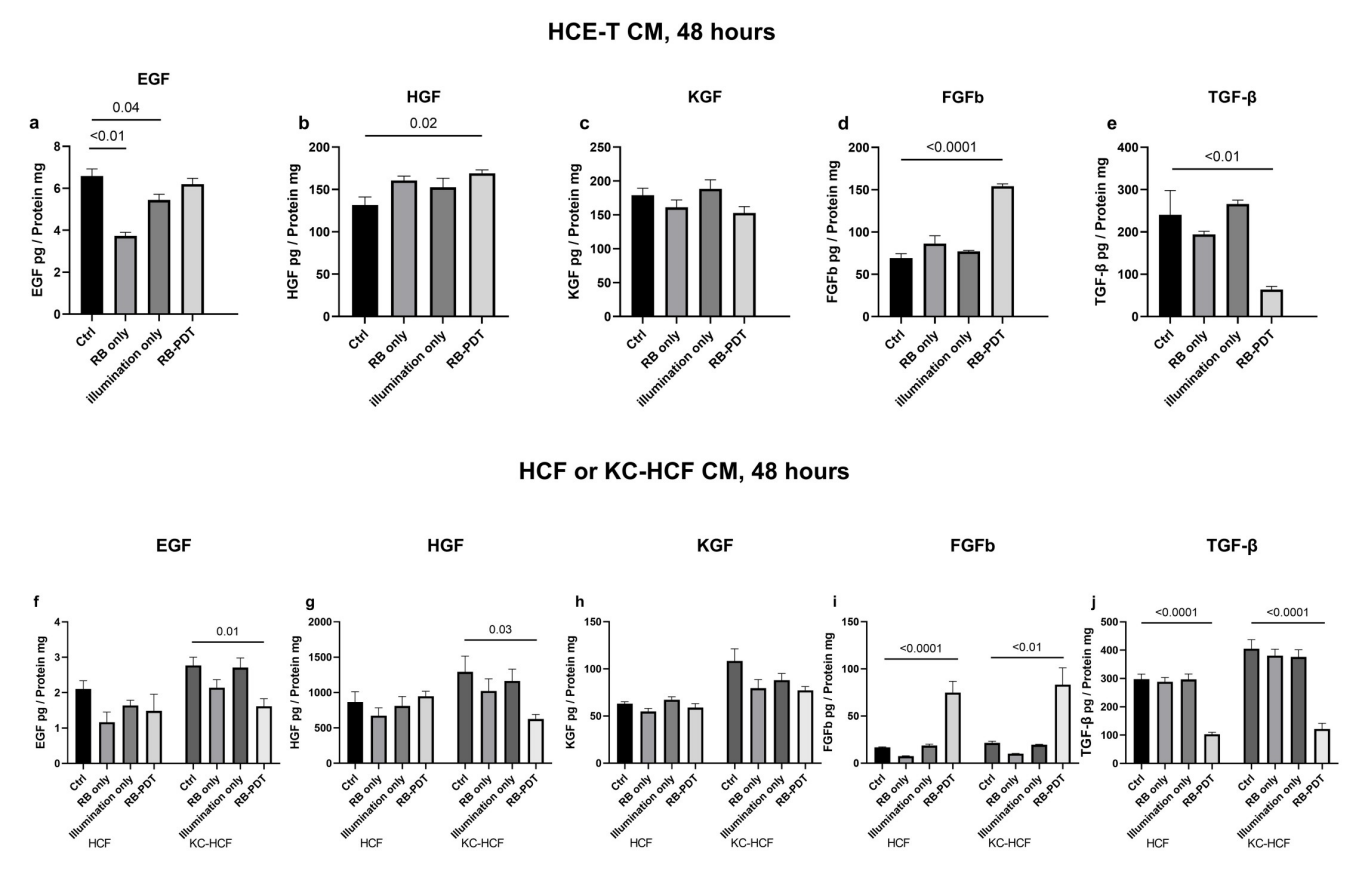
Growth factor concentration in 48 hours conditioned media (CM). Epidermal growth factor (EGF), hepatocyte growth factor (HGF) keratinocyte growth factor (KGF), fibroblast growth factor beta (FGFb) and transforming growth factor beta (TGF-β) concentration in human corneal epithelial cell (HCE-T) (n = 3), human corneal fibroblast (HCF) (n = 5) and human keratoconus fibroblast (KC-HCF) (n = 5) cell culture supernatant 48 hours after treatment, without scratching, were assessed by ELISA. Data are expressed as mean ± SEM. In HCE-T cell culture supernatant, 0.17 J/cm^2^ rose bengal photodynamic therapy (RB-PDT) significantly increased HGF and FGFb concentration (p≤0.02) and significantly decreased TGF-β concentration (p<0.01), compared to controls (Ctrl). In addition, rose bengal or illumination alone also significantly decreased EGF concentration in HCE-T cell culture supernatant, compared to controls (p≤0.04). In HCF cell culture supernatant, 0.17 J/cm^2^ RB-PDT significantly increased FGFb concentration (p<0.0001) and significantly decreased TGF-β concentration (p<0.0001), compared to controls. In KC-HCF cell culture supernatant, 0.17 J/cm^2^ RB-PDT significantly increased FGFb concentration (p<0.01) and significantly decreased EGF, HGF and TGF-β concentration (p≤0.03), compared to controls.

Growth factor concentration in HCE-T CM (without scratching) and in HCF and KC-HCF CM (without scratching) is shown at **Fig 6**.

In HCE-T CM, RB only and illumination only significantly decreased EGF concentration (p<0.01; p = 0.04) (**Fig 6A**). RB-PDT significantly increased HGF and FGFb concentration (p = 0.02; p<0.0001) and significantly decreased TGF-β concentration (p<0.01), compared to controls **(Fig 6B, 6D and 6E**).

In HCF CM, RB-PDT significantly increased FGFb concentration (p<0.0001), and significantly decreased TGF-β concentration (p<0.0001) compared to controls (**Fig 6I and 6J**). In KC-HCF CM, RB-PDT significantly decreased EGF, HGF and TGF-β concentration (p<0.01; p = 0.03; p<0.0001) and increased FGFb concentration (p<0.01), compared to controls (**Fig 6F, 6G, 6I and 6J**).

### 3.6 Growth factor concentration in the supernatant of scratched cell cultures, 48 hours after addition of CM

**Fig 7:**
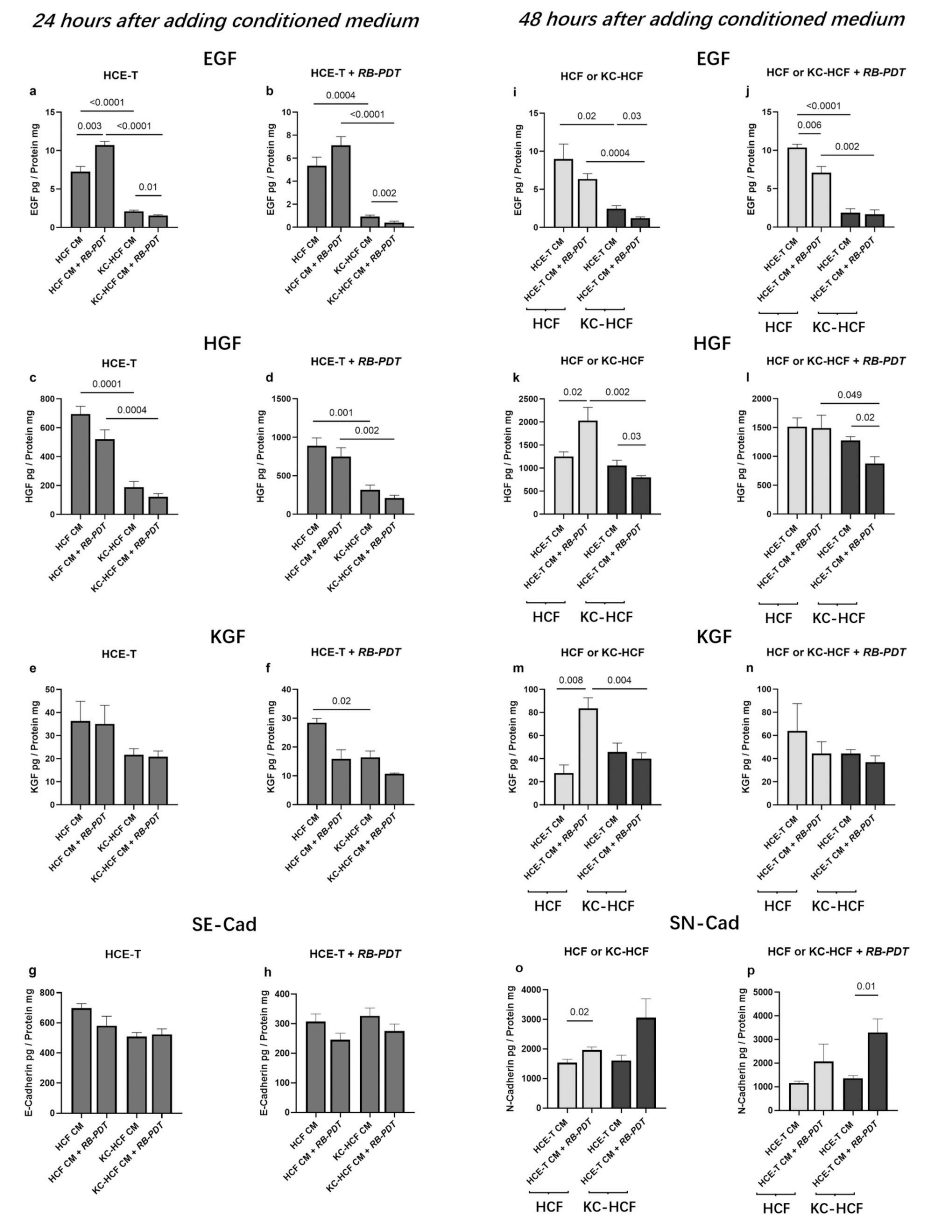
Epidermal growth factor (EGF), hepatocyte growth factor (HGF), keratinocyte growth factor (KGF), soluble E-Cadherin (SE-Cad) and soluble N-Cadherin (SN-Cad) concentration in human corneal epithelial cell (HCE-T) (n = 3), in human corneal fibroblast (HCF) (n = 5) and human keratoconus fibroblast (KC-HCF) culture supernatant (without or with RB-PDT of the scratched HCE-T, HCF or KC-HCF cultures), 24 or 48 hours after addition of different CM (a-p). CM has not been pooled, it has been used separately for different cultures. Data are presented as mean ± SEM. Significant p values (p<0.05) are indicated.

**Fig 7A–7P** shows EGF, HGF and KGF concentration in HCE-T, HCF and KC-HCF culture supernatant (without or with RB-PDT of the scratched HCE-T, HCF or KC-HCF cultures), 24 or 48 hours after addition of different CM.

In the supernatant of the scratched HCE-T cultures, without RB-PDT, EGF concentration was significantly higher using HCF CM following RB-PDT, than using HCF CM without previous RB-PDT (p = 0.003) (**Fig 7A**). However, both in the supernatant of the scratched HCE-T cultures with or without RB-PDT, EGF concentration was significantly lower using KC-HCF CM with RB-PDT, than with KC-HCF CM without RB-PDT (p = 0.01 and p = 0.002) (**Fig 7A and 7B**). In addition, both in the supernatant of scratched HCE-T cultures without and with previous RB-PDT, EGF and HGF concentration was significantly lower using KC-HCF CM without RB-PDT, than using HCF CM without RB-PDT (p≤0.001) (**Fig 7A–7D**). Both in the supernatant of scratched HCE-T cultures without and with previous RB-PDT, EGF and HGF concentration was significantly lower using KC-HCF CM after RB-PDT, than with HCF CM after RB-PDT (p≤0.002) (**Fig 7A–7D**).

KGF concentration in the supernatant of the scratched HCE-T cultures after RB-PDT, was significantly lower using KC-HCF CM without RB-PDT than using HCF CM without RB-PDT (p = 0.02) (**Fig 7F**). Nevertheless, KGF concentration in the supernatant did not differ significantly between any of the further scratched HCE-T cultures, using different CM (**Fig 7E and 7F**).

The **soluble E-Cadherin** (SE-Cad) concentration remained unchanged in the supernatant of the scratched HCE-T cultures, with or without RB-PDT, following the addition of HCF and KC-HCF CM (**Fig 7G and 7H**).

EGF concentration was significantly lower in the supernatant of the KC-HCF cultures without RB-PDT, than in the supernatant of the HCF cultures without RB-PDT, using HCE-T CM without RB-PDT (p = 0.02) (**Fig 7I**). Equally, EGF concentration was significantly lower in the supernatant of the KC-HCF cultures with RB-PDT, than in the supernatant of the HCF cultures with RB-PDT, using HCE-T CM, without RB-PDT (p<0.0001) (**Fig 7J**). In addition, EGF and HGF concentration was significantly lower in the supernatant of the KC-HCF cultures without RB-PDT, than in the supernatant of the HCF cultures without RB-PDT, using HCE-T CM after RB-PDT (p = 0.0004 and p = 0.002) (**Fig 7I and 7K**). Equally, EGF and HGF concentration was significantly lower in the supernatant of the KC-HCF cultures with RB-PDT, than in the supernatant of the HCF cultures with RB-PDT, using HCE-T CM after RB-PDT (p = 0.002 and p = 0.049) (**Fig 7J and 7L**).

In the supernatant of the scratched KC-HCF cultures, without RB-PDT, EGF and HGF concentration was significantly lower using HCE-T CM after RB-PDT, than using HCE-T CM without RB-PDT (p = 0.03 for both) (**Fig 7I and 7K**). In the supernatant of the scratched HCF cultures, with RB-PDT, EGF concentration was significantly lower using HCE-T CM after RB-PDT, than using HCE-T CM without RB-PDT (p = 0.006) (**Fig 7J**). In the supernatant of the scratched HCF cultures, without RB-PDT, HGF concentration was significantly higher using HCE-T CM after RB-PDT, than using HCE-T CM without RB-PDT (p = 0.02) (**Fig 7K**). In the supernatant of the scratched KC-HCF cultures, with RB-PDT, HGF concentration was significantly lower using HCE-T CM after RB-PDT, than using HCE-T CM without RB-PDT (p = 0.02) (**Fig 7L**).

In the supernatant of the scratched HCF cultures, without RB-PDT, KGF concentration was significantly higher using HCE-T CM after RB-PDT, than using HCE-T CM without RB-PDT (p = 0.008) (**Fig 7M**). KGF concentration was significantly lower in the supernatant of the KC-HCF cultures without RB-PDT, than in the supernatant of the HCF cultures without RB-PDT, using HCE-T CM after RB-PDT (p = 0.004) (**Fig 7M**).

In the supernatant of the scratched HCF cultures, without RB-PDT, SN-Cad concentration was significantly higher using HCE-T CM after RB-PDT, than using HCE-T CM without RB-PDT (p = 0.02) (**Fig 7O**). SN-Cad concentration was significantly higher in the supernatant of the KC-HCF cultures with RB-PDT, using HCE-T CM after RB-PDT, than using HCE-T CM without RB-PDT (p = 0.01) (**Fig 7P**).

## 4. Discussion

While RB-PDT has been used as a treatment for corneal pathologies *in vivo*, the effect of RB-PDT on human corneal epithelial cells and human corneal fibroblasts has not been analyzed in detail, yet.

In contrast to riboflavin-UVA-PDT (crosslinking), which is decreasing corneal fibroblast viability with 0.1% riboflavin concentration and 2 J/cm^2^ fluence [18], a 0.001% rose bengal concentration already resulted in decreased viability in corneal fibroblasts, using 0.17 J/cm^2^ fluence [3]. In a previous study, we further demonstrated that the minimum fluence during RB-PDT that decreases proliferation for HCE-T, HCF, and KC-HCF is 0.14 J/cm^2^, 0.17 J/cm^2^, and 0.14 J/cm^2^, respectively [3]. In this current study, considering the viability data and to maintain consistency in the preparation of conditioned medium, we used 0.17 J/cm^2^ fluence for RB-PDT. It is also worth noting that the results of the migration rate reflect the combined actions of migration and proliferation, both crucial for corneal wound healing [19, 20].

In this study, we observed a decrease in HCE-T, HCF, and KC-HCF migration rate 24 hours after 0.001% RB-PDT, with 0.17 J/cm^2^ fluence. While there was a decreasing trend, EGF and HGF concentrations in the cell culture supernatant did not change significantly across all cell types. However, KGF concentration significantly decreased in HCF and KC-HCF supernatant, 24 hours after the RB-PDT procedure.

KGF, a well-researched growth factor predominantly produced by corneal fibroblasts, plays a crucial role in epithelial-stromal interactions by regulating the motility and proliferation of corneal epithelial cells in a paracrine manner [21, 22]. However, its impact on corneal fibroblasts is debated [21, 23–25]. Wilson et al. suggested that KGF does not stimulate corneal stromal cell proliferation [22], while another study observed a significant increase in keratocyte density with different KGF concentrations [21]. Recent research by Cai et al. showed that KGF-2 promotes cell migration in rabbit corneal fibroblasts *in vitro* [25]. The decreased KGF level (**Fig 4C**) after scratching and RB-PDT in corneal fibroblast cultures may partly explain the reduced migration rate of HCF and KC-HCF after treatment.

Interestingly, green light illumination alone also resulted in KGF concentration decrease in HCF and KC-HCF supernatant, after 24 hours. While previous studies have reported damage from UVA and green light to human retinal cells [26], the specific impact of green light alone on corneal cells remains unexplored. Further investigation is needed to understand the mechanisms behind the decreased KGF concentration.

During corneal wound healing, interactions between the epithelium and stroma influence cell proliferation, migration, and differentiation, with various growth factors playing a role in this process [2]. To mimic these *in vivo* interactions, we introduced conditioned medium (CM) from RB-PDT-treated stromal cells into scratched corneal epithelial cell cultures, and *vice versa*.

In HCE-T cultures, using HCF CM after RB-PDT and in HCF cultures, using HCE-T CM after RB-PDT, the migration rate was significantly higher, than using HCF CM or HCE-T CM without RB-PDT (**Fig 5A and 5E**). Concurrently, the EGF concentration in HCE-T cultures using HCF CM after RB-PDT, and the HGF and KGF concentrations in HCF cultures using HCE-T CM following RB-PDT, were significantly higher, than those using HCF CM or HCE-T CM without RB-PDT (**Fig 7A, 7I and 7K**).

EGF plays a crucial role in modulating epithelial-stromal interactions, being secreted by either epithelial cells or stromal keratocytes [27, 28]. Within 30 minutes after wounding, the phosphorylation of EGF receptors in corneal epithelial cells has been observed [29]. Numerous reports suggest that EGF is essential for both epithelial cell proliferation and migration [24, 30]. The elevated EGF level in HCE-T cultures using HCF CM after RB-PDT may partly account for the observed differences in migration rates between groups after 24 hours (**Fig 7A**).

HGF, like KGF, is secreted by corneal fibroblasts, and both play an important role in facilitating the re-epithelialization process [31, 32]. In the case of an epithelial injury, epithelial cells release tumor necrosis factor-alpha (TNF-α), interleukin-1 (IL-1), and Fas ligand, which, in turn, stimulate the secretion of HGF and KGF by fibroblasts. These factors promote the proliferation and migration of corneal epithelial cells [23], potentially explaining the higher levels of HGF and KGF in HCF cultures after the use of HCE-T CM (**Fig 7I and 7K**). Furthermore, HGF is recognized as an anti-fibrotic factor in various organs, as it can counteract the TGF-β signaling pathway and promote the apoptosis of myofibroblasts [33].

To gain a better understanding of the composition of growth factors in the used CM, we also assessed EGF, KGF, HGF, FGFb and TGF-β levels in unscratched HCE-T CM, HCF and KC-HCF CM (**Fig 6**). We observed that growth factor levels in the aforementioned scratched cultures (**Fig 7**) did not entirely mirror the growth factor levels presented in CM, as there was no change in KGF level in unscratched HCE-T CM, nor EGF change in unscratched HCF CM (**Fig 6C and 6F)**. Therefore, we suspect that other substances in CM may participate in the regulation of these growth factors in the scratched cell cultures, such as IL-1α [34]. It is noteworthy that in CM of all cell types, FGFb levels increased and TGF-β levels decreased following RB-PDT, compared to controls. Both are crucial factors in corneal wound repair. An increase in FGFb facilitates the activation of epithelial cells and initiates cell migration, while a decrease in TGF-β suppresses corneal cell proliferation [34, 35]. Moreover, through the regulation of cell differentiation, TGF-β influences the wound healing outcome.

The process of corneal healing following injury involves the collaborative action of various cell types, including corneal epithelial cells, stromal fibroblasts, bone marrow-derived cells, immune cells, and cytokines [36, 37]. Myofibroblasts, characterized by the presence of α-smooth muscle actin (αSMA), play a crucial role in wound contraction and the secretion of extracellular matrix (ECM) components. Prolonged presence of myofibroblasts can lead to corneal opacity, also known as "haze". Wilson and Singh described that myofibroblasts can arise, both from corneal stromal fibroblasts and bone marrow-derived cells in response to TGF-β and PDGF [38, 39]. In this process, TGF-β can promote the entire developmental process of myofibroblast precursor cells to mature myofibroblasts by facilitating the expression of vimentin and αSMA. Simultaneous presence of TGF-β and PDGF significantly enhances αSMA expression, indicating the maturation of myofibroblast precursors. The blockage of these signals prevents the generation of myofibroblasts after corneal injury [31, 40–43]. Interestingly, a study has suggested that in the presence of heparin, FGFa and FGFb can reverse TGF-β mediated myofibroblast generation, leading to a transformation of myofibroblasts into fibroblasts [44]. In the present study, elevated FGFb levels and decreased TGF-β levels were observed in all types of CM after RB-PDT treatment. This observation may imply a potential beneficial role of RB-PDT in preventing excessive myofibroblast production.

In the healthy cornea, E-Cadherin serves as an adhesive molecule among epithelial cells. Meanwhile, N-Cadherin, functioning as an adhesion molecule for mesenchymal cells, is expressed in corneal fibroblasts and myofibroblasts [45]. Both E- and N-Cadherin belong to the Type I cadherin family, named for their dependence on calcium ions (Ca^2+^) [45]. During wound healing, Cadherin protein expression is intricately regulated due to cell migration and movement, achieving a dynamic balance [46, 47]. SE-Cad and SN-Cad have different functions compared to the full-length Cadherin. Nevertheless, in different cells types and tissues such as in endothelial cells, keratinocytes, pulmonary fibrosis, tumor, and inflammatory diseases, both SE-Cad and SN-Cad demonstrate a promoting effect on cell migration [47–52].

In this study, SE-Cad levels in scratched HCE-T cultures did not differ between groups (**Fig 7H and 7H**). Interestingly, in scratched HCF cultures, without RB-PDT, addition of RB-PDT treated HCE-T CM, increased SN-Cad level of the supernatant (**Fig 7O**), accompanied by an enhanced migration rate. However, in scratched, RB-PDT treated KC-HCF culture, the addition of RB-PDT treated HCE-T CM also increased SN-Cad concentration (**Fig 7H**), but it did not coincide with an increased migration rate. This might be related to increased susceptibility of KC-HCF to oxidative stress, which might slow down migration, compared to HCF [53]. In the scratched HCF cultures without the addition of HCE-T CM, we observed decreased SN-Cad concentration after RB-PDT, compared to untreated controls (**Fig 4D**), which was accompanied by a decreased migration rate (**Fig 3B**). These findings suggest that RB-PDT influences HCF adhesion and migration behavior by modulating SN-Cad levels, consequently affecting HCF migration rate.

Interestingly, the impact of PDT on migration and growth factor secretion of corneal cells differs depending on the photosensitizer/PDT type. Twenty-four hours-conditioned HCF and KC-HCF supernatant after 0.1% riboflavin CXL increased HCE-T migration [54]. In KC-HCF cultures, 24 hours after 0.1% riboflavin CXL, HGF, KGF, FGFb and TGF-β concentration did not differ significantly from untreated controls [18]. In another study, 24 hours after 100 nM chlorin e6 PDT of normal human fibroblasts, KGF secretion significantly decreased, while HGF, FGFb and TGF-β concentrations remained unchanged in the cell culture supernatant [55].

In conclusion, the migration rate of HCE-T, HCF, and KC-HCF is reduced 24 hours after RB-PDT. However, HCE-T migration is enhanced using HCF CM 24 hours after RB-PDT, and HCF migration rate is increased through HCE-T CM 48 hours following RB-PDT. The modulation of EGF, HGF, FGFb, TGF-β, and N-Cadherin secretion through RB-PDT may play a crucial role in corneal wound healing.

## Supporting information

S1 Fig The diagram of scratches. Three reference lines were drawn at the bottom of the wells of the 6-well plates with 5 mm distance. For each well, 4 different scratch areas have been photographically documented, along the previously drawn reference line.
(TIF)
