# Supplementary figures and images for: Human corneal epithelial cell and fibroblast migration and growth factor secretion after rose bengal photodynamic therapy (RB-PDT) and the effect of conditioned medium
Source: PLoS One. 2023 Dec 27;18(12):e0296022. doi: 10.1371/journal.pone.0296022 (PMC10752507; doi:10.1371/journal.pone.0296022)

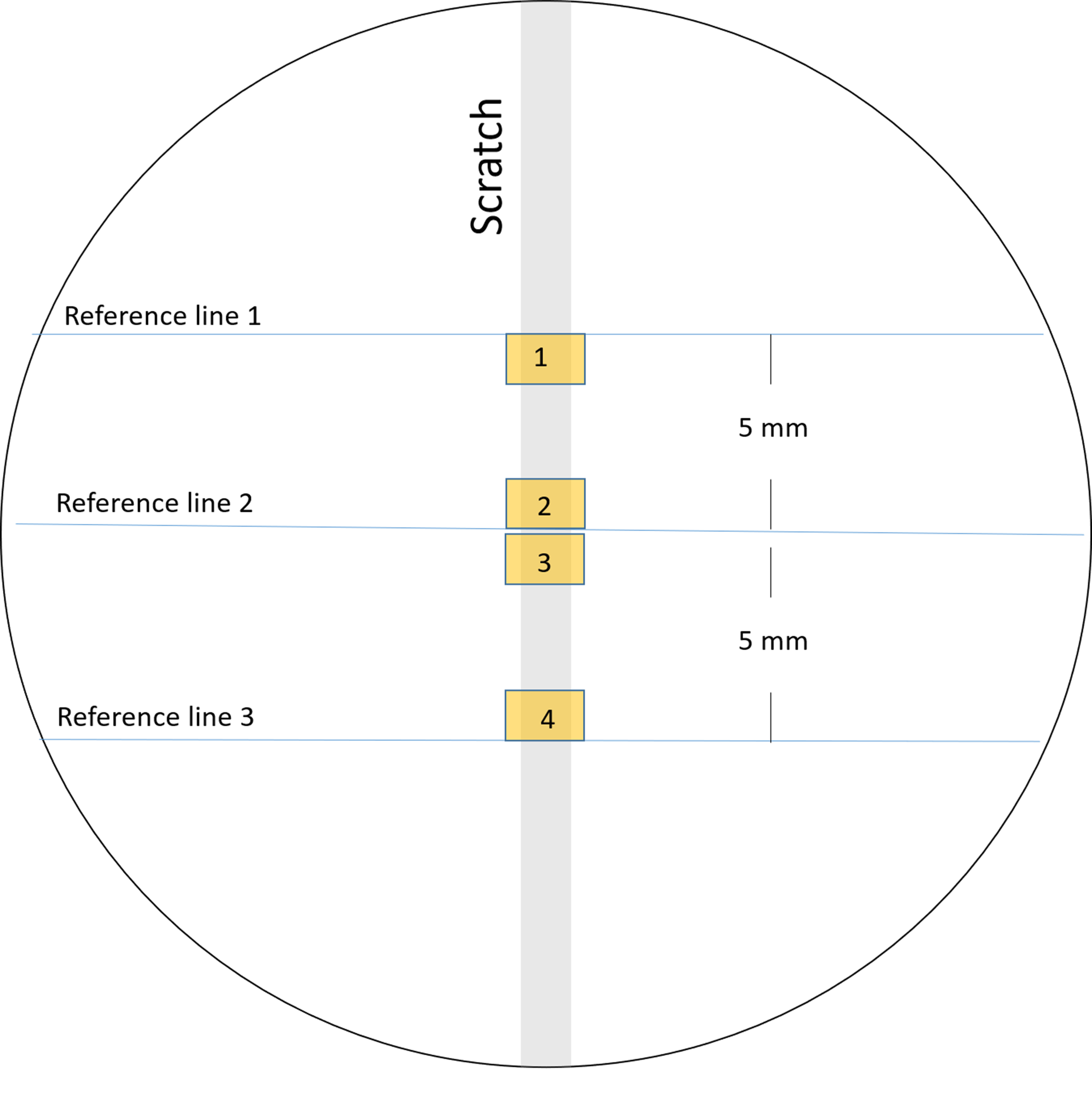

Supplement: S1 Fig — Three reference lines were drawn at the bottom of the wells of the 6-well plates with 5 mm distance. For each well, 4 different scratch areas have been photographically documented, along the previously drawn reference line. (TIF) [file pone.0296022.s001.tif]
